# The Effect of Morphine on Regional Cerebral Blood Flow Measured by ^99m^Tc-ECD SPECT in Dogs

**DOI:** 10.1371/journal.pone.0109680

**Published:** 2014-10-08

**Authors:** Antita Adriaens, Kathelijne Peremans, Tim Waelbers, Eva Vandermeulen, Siska Croubels, Luc Duchateau, André Dobbeleir, Kurt Audenaert, Jos Eersels, Simon Vermeire, Bart De Spiegeleer, Ingeborgh Polis

**Affiliations:** 1 Department of Medicine and Clinical Biology of Small Animals, Faculty of Veterinary Medicine, Ghent University, Merelbeke, Belgium; 2 Department of Medical Imaging of Domestic Animals, Faculty of Veterinary Medicine, Ghent University, Merelbeke, Belgium; 3 Department of Pharmacology, Biochemistry and Toxicology, Faculty of Veterinary Medicine, Ghent University, Merelbeke, Belgium; 4 Department of Physiology and Biometry, Faculty of Veterinary Medicine, Ghent University, Merelbeke, Belgium; 5 Department of Psychiatry and Medical Psychology, Faculty of Medicine and Health Sciences, Ghent University, Ghent, Belgium; 6 Nuclear Medicine and PET research, VU University Medical Centre, Amsterdam, The Netherlands; 7 Drug Quality & Registration (DruQuaR) group, Faculty of Pharmaceutical Sciences, Ghent University, Ghent, Belgium; Nathan Kline Institute and New York University School of Medicine, United States of America

## Abstract

To gain insights into the working mechanism of morphine, regional cerebral blood flow (rCBF) patterns after morphine administration were assessed in dogs. In a randomized cross-over experimental study, rCBF was estimated with ^99m^Tc-Ethylcysteinate Dimer single photon emission computed tomography in 8 dogs at baseline, at 30 minutes and at 120 minutes after a single bolus of morphine. Perfusion indices (PI) in the frontal, parietal, temporal and occipital cortex and in the subcortical and cerebellar region were calculated. PI was significantly decreased 30 min after morphine compared to baseline in the right frontal cortex. The left parietal cortex and subcortical region showed a significantly increased PI 30 min after morphine compared to baseline. No significant differences were noted for the other regions or at other time points. In conclusion, a single bolus of morphine generated a changing rCBF pattern at different time points.

## Introduction

Morphine, a mu opioid receptor agonist, is a frequently used potent analgesic in human and veterinary medicine. Its pharmacological effect depends on peripheral and supraspinal mu opioid receptor (MOR) activation (for review see [1]). Cerebral MORs are widely distributed throughout the brain and modulate the transmission and perception of painful stimuli [2].

Numerous studies have reported on different aspects of the influence of various opioids on cerebral hemodynamics, both in animal models and in man [3]–[10]. However, the effects of opioids on cerebral blood flow (CBF) remain unclear due to the variable results [3]–[10]. In dogs, only few have reported on the hemodynamic effects of morphine on the brain, usually reporting global cerebral blood flow changes with varying results [4], [11]–[16]. These discrepancies are probably caused by differences in study design, imaging technique, species, opioid choice, dose and route of administration.

It is important to determine whether regional CBF (rCBF) differences occur in order to better understand how drugs influence cerebral activity and, therefore, to gain insight on their mechanism of action. Particularly in the case of mu opioid analgesics this is interesting since their side effects (dependence, tolerance, addiction) are thought to be mainly caused by supraspinal mechanisms in which the MOR plays a key role [17].

Single photon emission computed tomography (SPECT) imaging using ^99m^Tc-Ethylcysteinate Dimer (^99m^Tc-ECD) is a valid method for rCBF evaluation in dogs [18]. As rCBF is correlated to regional brain activity [19], it can be used to study the effects of different drugs on the canine brain [20]. The objective of the present study was to examine the effects of a single dose of morphine on rCBF in dogs. Since opiate action is not only dose, but also time dependent [21], [22], SPECT scans were performed at different time intervals after morphine administration.

## Materials and Methods

### Animals

Eight female neutered Beagle dogs (age 4.22±0.35 years, weight 8.58±0.87 kg (mean ± standard deviation, SD)) were used for this study. None of the dogs had a history of major disease or neurological disorder. The study was approved by the Ghent University Ethical Committee (EC 2011-130). All guidelines for animal welfare, imposed by the Ethical Committee, were strictly respected. All procedures were conducted under veterinarian supervision. The animals were housed in pairs in pens and received environmental enrichment. Food was provided once daily and water was given ad libitum.

### Study Design

To investigate the influence of a single rapid bolus of morphine (0.5 mg/kg intravenously, Morphine Hydrochloride, Sterop, Belgium) on rCBF, all dogs were scanned 3 times in a randomized cross-over study design. The 3 SPECT scans consisted of a baseline scan (no morphine, condition ‘Baseline’), a scan where ^99m^Tc-ECD was injected 30 minutes after morphine (i.e. T30, condition ‘MOR30’) and a scan where ^99m^Tc-ECD was injected 120 minutes after morphine (i.e. T120, condition ‘MOR120’). The time between scans was 3 weeks. All scans were performed following the same anesthetic and image acquisition protocol. Food, but not water, was withheld for 12 hours before each scan, to reduce the risk of aspiration during anesthesia. To determine morphine plasma and cerebrospinal fluid (CSF) concentrations 2 hours after morphine administration, 5 mL of blood were collected from the jugular vein (in a heparinized blood tube) and approximately 2 mL of CSF were collected by cisternal puncture from all dogs under anesthesia after the MOR30 scan. Blood was centrifuged at 3500 rpm for 5 minutes after which plasma was collected. All samples were kept at −20°C until analysis was performed.

### Tracer

To evaluate rCBF ^99m^Tc-ECD (Neurolite, Bristol-Myers Squibb Medical Imaging), prepared according to the manufacturer's instructions, was used. ^99m^Tc-ECD passes the blood-brain-barrier in its lipophilic form after which it is enzymatically transformed by intracellular esterases to a hydrophilic form that remains trapped in the cell. It rapidly reaches plateau values in the brain from 5 min onwards thus providing a fixed image of the rCBF practically at the moment of ^99m^Tc-ECD injection. Optimal image acquisition is achieved between 15 and 40 min after tracer injection [23]. In the present study, image acquisition was started 40 min after injection of the tracer. ^99 m^Tc-ECD was administered intravenously: 747.91±30.22 MBq, 789.16±47.02 MBq and 751.61±40.33 MBq in the Baseline, MOR30 and MOR120 condition, respectively.

### Anesthetic Protocol

General anesthesia is required for SPECT imaging in animals. Prior to the study, an intravenous catheter was placed to ensure correct drug administration. To avoid the influence of anesthetic drugs on rCBF, the administration of the anesthetics was done after the plateau for ^99m^Tc-ECD was reached in the canine brain. All dogs were sedated prior to general anesthesia with intramuscular dexmedetomidine 375 µg/m^2^ body surface area (Dexdomitor, Orion Corporation, Espoo, Finland). After induction with intravenous propofol (Propovet, Abbott Laboratories, Queenborough, United Kingdom), the dogs were intubated and anaesthesia was maintained with isoflurane (Isoflo, Abbott Laboratories) in oxygen. Acquisition started 10 minutes after induction of anesthesia. The dogs were monitored during anesthesia and were mechanically ventilated by intermittent positive pressure ventilation to maintain eucapnia, i.e. end tidal CO_2_ within 35–45 mmHg, in order to limit the influence of the PaCO_2_ on cerebral perfusion. After the SPECT scan, the dogs were monitored during the recovery period. The procedure was well tolerated by all animals. The administration of morphine induced a short period of panting and mild sedation in some dogs, as was expected. No other side effects were noted during the experiments.

### Image Acquisition

Image acquisition started 10 minutes after induction of anesthesia, i.e. 40 min after ^99m^Tc-ECD injection. Circular orbit SPECT scans were performed using a triple head gamma camera (Triad Trionix, Twinsburg, OH, USA) equipped with ultrahigh resolution parallel hole collimators (tomographic resolution, 8 mm full width half maximum). For each acquisition, 120 projection images were obtained on a 128×128 matrix using a step-and-shoot mode (360° angular sampling, 3° steps, 10 s per step), with a total scanning time of 20 minutes. A 20% window setting, centered symmetrically on the photon energy of ^99m^Tc (140keV), was used. During scanning, dogs were positioned in ventral recumbence with fixation of the head in a preformed cushion, to prevent individual positioning artifacts. Camera and table positioning were recorded to ensure optimal intraindividual comparison.

### Image Processing

The images were reconstructed using filtered backprojection and post-filtering with a Butterworth filter (cut-off, 1.6 cycles/cm, order 5). Pixel size was 1.72 mm. Average whole brain counts registered in the blank scan, MOR 30 and MOR 120 scans were 995830±305258, 1183867±314715 and 1017598±418056 respectively.

The emission data were fitted to a template, which provides anatomical references and a predefined region map, with BRASS software (Brain Registration and Automated SPECT Semiquantification, Hermes Gold (version 2.10), Nuclear Diagnostics, Stockholm, Sweden). This software allows scaling, rotating, and translating the acquired images in all 3 dimensions in order to fit them to this template. It was originally developed for humans but the template and regions were created in house for the use in dogs [24]. Using this template with the predefined region map, allows for a volume-of-interest (VOI) approach. The following VOIs were included: right and left frontal, temporal, parietal and occipital cortices, as well as the subcortical and cerebellar region. Perfusion indices (PI) for the different VOIs were calculated as the mean count per voxel in each VOI normalized to the mean total brain count per total voxel.

### Morphine Concentrations

Plasma and CSF concentrations of morphine were determined using a validated liquid chromatography-tandem mass spectrometry (LC-MS/MS) method, as previously described by Gadeyne et al. [25]. Quality control (QC) and blank samples were analyzed together with each batch of incurred samples to check the extraction and LC-MS/MS procedure. All QC, blank and calibration curve samples were prepared in pooled drug-free dog plasma. The limit of detection of the method was calculated to be 1.27 ng/mL based on a signal-to-noise ratio of 3/1. The accuracy and precision were determined at 10.0 and 50.0 ng/mL in dog plasma (n = 6). The accuracy fell within the range of −20% to +10%, and precision was also within the maximum RSD values as set by the EU [26]. The limit of quantification (LOQ) was defined as the lowest concentration in dog plasma for which the method was validated with an accuracy and precision that fall within EU recommended ranges and was set at 5.0 ng/mL. Plasma and CSF concentrations below the LOQ were not included in the data analysis.

### Statistical Analysis

To compare PI means between the three conditions, a mixed model was fitted with condition (Baseline, MOR30 and MOR120) as fixed effect and dog as random effect. Left-right differences for the cortical regions were calculated by means of non-parametric Wilcoxon Signed Ranks Test. Correlation between morphine plasma and CSF concentration was investigated by means of Kendall Tau b correlation coefficients.

Significance was set at *P*<0.05. All data are presented as mean ± SD.

## Results

Table 1 depicts PIs for all VOIs in the different conditions. Mean PIs of the right frontal cortex, left parietal cortex and the subcortical region differed significantly between conditions. Pairwise comparison revealed that morphine administration only altered the rCBF in those regions compared to the baseline condition at T30 (*P* = 0.047, 0.021 and 0.006 respectively). No significant differences were found when comparing Baseline to MOR120 or MOR30 to MOR120.

**Table 1:** Perfusion indices for the different conditions (Baseline, MOR30 and MOR120) in all volumes of interest (VOI) in 8 dogs (mean ± SD).

VOI	Baseline	MOR30	MOR120	*P*
LF	1.12±0.05	1.12±0.06	1.12±0.04	0.89
RF	1.12±0.02	1.06±0.07	1.11±0.04	0.04*
LT	0.86±0.02	0.90±0.07	0.89±0.05	0.23
RT	0.85±0.05	0.85±0.04	0.90±0.05	0.09
LP	1.12±0.07	1.21±0.06	1.19±0.07	0.02*
RP	1.11±0.04	1.15±0.10	1.20±0.09	0.08
LO	1.08±0.07	1.09±0.13	1.00±0.07	0.12
RO	1.07±0.05	1.07±0.14	1.02±0.08	0.36
Subcortical	1.24±0.03	1.29±0.04	1.26±0.05	0.01*
Cerebellum	1.05±0.02	1.04±0.06	1.00±0.06	0.14

Data compared with mixed model. *Pairwise comparisons revealed a significant difference between Baseline and MOR30 in RF, LP and SC (*P* = 0.047, 0.021 and 0.006 respectively). LF, left frontal; RF, right frontal; LT, left temporal; RT, right temporal; LP, left parietal; RP, right parietal; LO, left occipital; RO, right occipital.

Table 2 shows the mean left-right differences for the frontal, temporal, parietal and occipital cortices. Significant left-right differences were found in the forntal cortex for all conditions and in the occipital cortex in the MOR30 condition.

**Table 2:** Differences (mean ± SD) between perfusion indices of left and right cortical regions in 8 dogs in the different conditions (Baseline, MOR30 and MOR120).

	Baseline	MOR30	MOR120
LF-RF	0.00±0.05*	0.05±0.05*	0.01±0.05*
LT-RT	0.01±0.04	0.05±0.07	0.00±0.07
LP-RP	0.02±0.07	0.06±0.06	−0.01±0.09
LO-RO	0.01±0.04	0.01±0.05*	−0.01±0.07

*Significant difference between left and right region (*P*<0.05).

Two CSF samples had morphine concentrations below the LOQ and were not used for further analysis. Morphine plasma and CSF concentrations 2 hours after morphine administration were 13.50±3.75 ng/mL and 17.52±1.32 ng/mL, respectively (individual concentrations are presented in Table 3). There was no significant correlation between morphine concentrations in plasma and CSF (Kendall Tau correlation coefficient 0.43 and *P* = 0.14).

**Table 3:** Morphine concentrations (ng/mL) in plasma and CSF in 8 dogs 120 minutes after intravenous morphine administration at a dose of 0.5 mg/kg.

Dog	Plasma	CSF
1	16.9	17.6
2	12.6	19.1
3	18.0	18.5
4	10.1	15.7
5	7.2	<LOQ
6	15.3	18.0
7	16.3	<LOQ
8	11.6	16.2

LOQ: Limit of quantification.

## Discussion

The current study investigated changes in rCBF after morphine administration in dogs at two different time points. In non-painful dogs, a single dose of morphine induced rCBF changes in the frontoparietal cortex after 30 minutes compared to baseline values. We found a decrease in blood flow in the frontal cortex and an increase in blood flow in the parietal cortex. These brain regions are known to be involved in the complex mechanism of pain perception, i.e. the cognitive and affective-motivational aspects of nociception (for review see [27]). The parietal lobes are part of the somatosensory systems, playing a role in not only the perception of painful and non-painful stimuli, but also in their modulation. Additionally, a study in rats provided evidence in the role of the somatosensory areas in the induction of morphine addiction, where lesions in these regions would impair conditioned place preference [28].

Additionally, an increased CBF was found in the subcortical region which includes structures important for pain modulation such as the thalamus and the periaqueductal gray (for review see [29]). Alterations in rCBF reflect alterations in brain activity, i.e. increased brain activity requires increased blood supply and vice versa [19]. It is therefore not surprising to find rCBF changes in regions involved in the modulation and perception of pain after the administration of a potent analgesic such as morphine. Furthermore, the present results are in line with a study in humans on the effects of opioids and provoked pain on rCBF [9]. In that study it was found that regions involved in the modulation of pain were activated, whereas regions that are involved in the perception of pain showed a decreased rCBF after opioid administration [9]. In dogs, the effects of opioids on global CBF have been investigated and decreased as well as increased global CBF have been reported [12], [15], [16].

Remarkably, we only found a significant difference in rCBF between MOR30 and Baseline and not between MOR120 and Baseline. In a study in dogs using remifentanil, decreased blood flow and decreased EEG activity were described in the cortex, hippocampus and caudate nucleus [4]. These changes recovered to baseline levels after 30 minutes [4]. Since morphine is a longer acting opioid, with a halftime of approximately 60 minutes in dogs at the same intravenous dosage of 0.5 mg/kg [30], it is possible that a comparable return to baseline brain activity occurs at a later time point.

The morphine plasma concentrations at T120 were 13.50±3.75 ng/mL, these are comparable to the plasma concentrations needed to achieve 50% of maximal effect obtained in a pharmacodynamic study in dogs using 1 mg/kg morphine IV, i.e. 13.92±2.39 ng/mL [31]. In that study it was also reported that a significant analgesic level was present for only 2.8±0.6 hours [31]. In a clinical setting on the other hand, morphine is usually administered every 4 hours to maintain proper analgesia. Indeed, recommended morphine dosages in dogs range from 0.1 to 0.5 mg/kg intravenously, with lasting effects up to 3 to 4 hours [32]. In a study in dogs on the pharmacokinetics of morphine, CSF concentrations at T120 were higher than plasma concentrations, as is the case in our study for most dogs and the range of CSF concentrations at T120 are also comparable to our results (Table 3) [33]. It was additionally described that morphine appears to accumulate in CSF over time after systemic administration, since it has a longer halftime in CSF (121±6 min) compared to plasma (75±5 min) and the CSF-to-plasma concentration gradient increases progressively [33]. Therefore, a single dose of morphine will have plasma concentrations that decrease more rapidly than CSF concentrations, leading to an underestimation of the effective plasma concentrations after the administration of a single dose of morphine.

Taken together with our results, at T120 after morphine administration, we know that plasma and CSF concentrations are present and effective, however, no changes in blood flow – and thus brain activity – are detectable at this time point. This could possibly suggest that morphine exerts its effect at a different level at this time point. On the one hand, changes in the serotonergic neuroreceptor system have been found in a previous study, possibly indicating that the downstream effects of morphine are more important for its effect [34]. On the other hand, a peripheral action (spinal or at the periphery) rather than a supraspinal action could be responsible for the effect of morphine at a later time point. This possibly insinuates a time-dependent localization of the analgesic effect of morphine, which would be interesting for further investigation in future studies.

Differences in perfusion were found when comparing the left and right frontal cortices in all conditions. Seeing as this is consistent throughout the conditions, a specific effect due to the administration of morphine is unlikely. For the found elevated blood flow in the left occipital region compared to the right occipital region in the MOR30 condition we do not have a clear explanation.

As for the effects of opioids on cerebral hemodynamics in dogs, it is rather unlikely that changes in systemic cardiovascular parameters would cause regional alterations in CBF. Next to the fact that morphine at clinically relevant doses has limited influence on systemic cardiovascular parameters such as heart rate and blood pressure [35], [36], circulatory changes would be expected to cause more global CBF changes that cannot be properly assessed with the technique used in the present study.

Additionally, if proper ventilation is assured (opioids can cause respiratory depression), the effects of opioids on cerebral hemodynamics are limited when used at clinical doses [37].

Several explanations for the alterations in rCBF caused by opioids have been suggested. The observed rCBF changes are most likely the result of changes in neuronal activity since MOR activation in itself requires increased energy demand, thus increasing blood flow. An effect of opioids on cerebral vasculature has also been proposed, either direct or indirect [38], [39]. However, this would imply that changes in rCBF are only related to MOR density, which is not always the case due to the widespread distribution of cerebral MORs [2], [5], [40], [41] and the widespread influence of opioids on different neurotransmitter systems [42]–[44].

## Conclusion

The present study found an altered rCBF pattern 30 minutes after morphine compared to baseline values. Pain is a complex phenomenon and unraveling the working mechanism of analgesics such as opioids appears to be equally challenging, involving various cerebral regions. On top of this, the possibly time-dependent rCBF pattern found after morphine raises new questions on the correct interpretation of data and the comparison with other results.
